# Saccadic Compression of Rectangle and Kanizsa Figures: Now You See It, Now You Don't

**DOI:** 10.1371/journal.pone.0006383

**Published:** 2009-07-27

**Authors:** Atsushi Noritake, Bob Uttl, Masahiko Terao, Masayoshi Nagai, Junji Watanabe, Akihiro Yagi

**Affiliations:** 1 Brain Science Institute, Tamagawa University, Tokyo, Japan; 2 Psychology Department, Mount Royal College, Calgary, Alberta, Canada; 3 Graduate School of Humanities, Kwansei Gakuin University, Hyogo, Japan; 4 National Institute of Advanced Industrial Science and Technology, Ibaragi, Japan; 5 PRESTO Japan Science and Science and Technology Agency, Kanagawa, Japan; 6 Department of Psychology, Kwansei Gakuin University, Hyogo, Japan; University of Leuven, Belgium

## Abstract

**Background:**

Observers misperceive the location of points within a scene as compressed towards the goal of a saccade. However, recent studies suggest that saccadic compression does not occur for discrete elements such as dots when they are perceived as unified objects like a rectangle.

**Methodology/Principal Findings:**

We investigated the magnitude of horizontal vs. vertical compression for Kanizsa figure (a collection of discrete elements unified into single perceptual objects by illusory contours) and control rectangle figures. Participants were presented with Kanizsa and control figures and had to decide whether the horizontal or vertical length of stimulus was longer using the two-alternative force choice method. Our findings show that large but not small Kanizsa figures are perceived as compressed, that such compression is large in the horizontal dimension and small or nil in the vertical dimension. In contrast to recent findings, we found no saccadic compression for control rectangles.

**Conclusions:**

Our data suggest that compression of Kanizsa figure has been overestimated in previous research due to methodological artifacts, and highlight the importance of studying perceptual phenomena by multiple methods.

## Introduction

Several lines of evidence demonstrate that observers misperceive the location of points within a visual scene presented at about the time of a saccade [1]. Two kinds of misperceptions have been reported in the literature: saccadic mislocalization and saccadic compression. In saccadic mislocalization, all the points of visual space are shifted in the same uniform direction. To illustrate, Honda [2] required participants sitting in complete darkness to locate a single flash of light presented in various locations around the saccadic target shortly before, during, or after the saccade. He reported that a flash presented briefly before a saccade is perceived as shifted in the direction of the saccade. However, if the flash is presented following the saccade, the direction of the shift gradually reverses and the flash is perceived as shifted in the direction opposite the saccade, with the maximum shift occurring about 50 ms after the saccade onset. Importantly, the flash is shifted the same way regardless of its actual location relative to the saccade target. In contrast, in saccadic compression, the points of visual space are shifted towards the saccade target. Ross, Morrone, and Burr [3] were the first to report that when observers are provided with a visual reference, such as a ruler, throughout the duration of a trial, the apparent location of the flash presented around the time of saccades shifts toward the saccade target as if the visual space surrounding the saccadic target was compressed. Thus, whether the points of visual space are mislocalized along the same uniform direction or whether they are perceived as compressed towards the saccadic target seems to depend crucially on provision of a visual reference [4]. To explain these findings, Morrone, Ross, and Burr [5] proposed a mathematical model that models compression of each point in the visual space by the same mathematical equation independently of compression for any other point.

Contrary to Morrone et al.'s [5] proposal, more recent studies have revealed that compression of visual space is dependent upon the type of visual stimulus. Specifically, Matsumiya and Uchikawa [6] compared the apparent width of four vertical bars presented just prior to saccade onset with the apparent width of a rectangle, and found compression of the vertical bars but not the rectangle. Moreover, Matsumiya and Uchikawa demonstrated that compression depends critically on whether the individual elements are perceived as a single object. Using the same saccadic task, they presented participants with a solid rectangle, an outline rectangle (composed of disk elements), and three outline vertical bars (also composed of disk elements). Although their data showed some individual variability in the degree of compression, in general, the rectangle was not compressed, the outline rectangle was subject to some but relatively small compression, and the outline bars were substantially compressed. In combination, these results suggest that saccadic compression does not occur for a constellation of elements perceived as a single object, at least when such object perception is induced by element proximity, but that whenever several objects are presented the location of each shifts towards the saccade goal.

However, we can induce perception of discrete elements as a single object not only through element proximity but also by other means. To illustrate, the Gestalt principle of closure suggests that we perceive familiar figures with gaps (i.e., constellation of discrete elements) as complete figures or objects. Perhaps one of the best-known illustrations of closure is the Kanizsa figure [7] that induced perception of an object, for example, a triangle or a rectangle, by a circular disk with wedges cut out (i.e., Pac-man) and oriented in such a way that extension of the wedges forms the object. Thus, the Kanizsa figure induces observers to see contours even though “they are not there” since the luminance or colors between a figure and the background is the same. If any discrete elements perceived by observers as a single object are not compressed, we should see no compression with the Kanizsa figure. However, if the critical element is proximity, rather than perception as a single object, we should see compression with large Kanizsa figures but not with small Kanizsa figures. Accordingly, the question whether a Kanizsa figure compresses has important implications about the nature of processes involved in saccadic compression.

**Figure 1:**
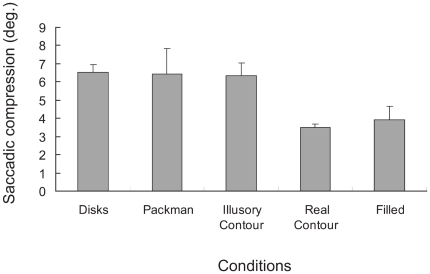
Averaged magnitude of horizontal saccadic compression for participants in Sogo and Osaka's Experiment 1. Bars indicated the averaged magnitude of the saccadic compression for all conditions: Disks, Packman, Illusory contour, Real contour, and Filled figures. Error bars indicated standard deviation of the compression among their three participants. The figure highlights that Sogo and Osaka observed substantial horizontal saccadic compression even for the control figures: the real contour and filled contour conditions.

Sogo and Osaka [8] recently published the first study using Kanizsa figures to investigate aspects of visual stimuli that influence saccadic compression. Participants in their study saw a stimulus flash about the time of saccade onset; 1500 ms following the saccade offset, they were presented with a probe – a test figure of the same shape and size but either shortened or lengthened in the horizontal dimension. The participants' task was to adjust the horizontal dimension of this probe so that it matched the presented stimulus. Sogo and Osaka reported that Kanizsa figures compressed horizontally, suggesting that the re-mapping process occurs prior to the processes constructing illusory contours and that element proximity but not closure affects saccadic compression. However, their results need to be viewed with caution because of several design features of their study. The methods allow participants to use two distinct strategies: adjusting the length of the probe to the *length of remembered figure* or adjusting the length of the probe so that the probe matched the *shape of the remembered figure*. If participants match the probe to the remembered shape and if the vertical dimension of the figure is either misperceived (e.g., compressed) or misremembered (e.g., taller or shorter), Sogo and Osaka's data may underestimate or overestimate the degree of horizontal compression. Several lines of evidence highlight this possibility. First, Kaiser and Lappe [9] showed that saccadic compression occurs in both vertical and horizontal dimensions, at least under some circumstances, and that the vertical dimension (i.e., the dimension orthogonal to the saccade direction) may compress as much as the horizontal dimension. Second, Sheth and Shimojo [10] showed that inserting even a short delay (e.g., several hundred ms) between the presentation of a target and a probe increases observer *reported*, as opposed to actual, visual compression. Consistent with these concerns, Sogo and Osaka reported an odd finding: they found substantial horizontal compression even for the control rectangle figures, but offered no explanation for it. Figure 1 summarizes Sogo and Osaka's findings and highlights that they observed substantial compression for all of their stimuli including control rectangles that should not be compressed. In turn, Sogo and Osaka's data are difficult to interpret because there is no way to assess the influence of participants' strategies and memory on their findings.

We used Kanizsa figure to gain insight into the nature of processes involved in saccadic compression. More specifically, our aims were to obtain unequivocal evidence, free of interpretive difficulties, showing whether saccadic compression occurs with Kanizsa figure stimuli, to determine at which stage of processing such compression occurs, to investigate whether saccadic compression is dependent on proximity or closure. As a starting point of our design and based on extensive prior evidence, we took for granted that visual space is not extended just prior to saccadic movement, neither in the horizontal nor vertical dimension (e.g., [3], [4], [6], [11]). Moreover, to avoid the interpretive difficulties discussed above and to obtain more objective data, uncontaminated by participants' memory and length estimation abilities, we employed a widely used two-alternative forced choice method (2-AFC) that required participants to decide whether the horizontal or vertical length of stimulus was longer immediately after the saccade offset. To increase the generalizability of our findings and to assess whether horizontal vs. vertical compression is relatively larger, we varied both horizontal (Experiment 1a and 1b) and vertical (Experiment 2) dimensions of the stimulus target.

### Overview of Experiments and Results

#### Experiment 1a

In Experiment 1a, we investigated horizontal saccadic compression for Kanizsa and control rectangle figures presented in two different sizes (small, large). Participants were presented with Kanizsa and control rectangles and had to decide whether the horizontal length of each stimulus was longer using the two-alternative force choice method. To control for any possible perceptual biases and to assess participants' ability to estimate relative length of horizontal vs. vertical dimension of critical figures (Kanizsa, rectangle), participants were also required to make the two-alternative force choice judgments about the stimuli while their eyes remained stationary (fixation condition). Relative to the fixation condition, Experiment 1a revealed horizontal saccadic compression for large Kanizsa figures but not for large rectangle stimuli, small Kanizsa stimuli, or small rectangle stimuli.

#### Experiment 1b

To find out whether the illusory contours are responsible for preventing saccadic compression of the small Kanizsa figure, we repeated Experiment 1a with three different small figures: rectangle, Kanizsa, and disk-rectangle. The disk-rectangle was similar to the Kanizsa figure except that the Pac-men – the illusory shape inducers – were replaced by disks (filled circles). If illusory contours are critical for prevention of saccadic compression, then saccadic compression would occur in the disk-rectangle but not in the Kanizsa conditions. In contrast, if proximity of the figure elements is critical, then saccadic compression would not occur in either condition. Experiment 1b revealed no horizontal saccadic compression for any of the small figures.

#### Experiment 2

As noted in the introduction, Kaiser and Lappe [9] showed that when a single point flash was presented at more than 20 degree eccentricities from the fixation point just prior to saccade onset, saccadic compression occurs in both vertical and horizontal dimensions, and that compression in the vertical dimension (i.e, the dimension orthogonal to saccade direction) is as large as compression in the horizontal dimension. However, no previous study has investigated whether vertical compression occurs with complex stimuli such as rectangles or Kanizsa figures. Accordingly, in Experiment 2 we varied vertical dimensions of rectangles and Kanizsa figures to find out whether vertical compression also occurs with these complex stimuli. Experiment 2 revealed no evidence of vertical saccadic compression for neither Kanizsa figures nor rectangle figures.

## Methods

### Experiment 1a: Method

#### Participants & Design

Four women and nine men (mean age 24.3 years, range 21 to 24 years) participated in Experiment 1a. One participant was an author of the present study but other participants did not know the purpose of the experiment. The design had three within-subject factors: stimulus type (Kanizsa, rectangle), size (small, large), and eye movement condition (saccade, fixation). The experiment was approved by Research Ethics Committee of the Kwansei Gakuin University and all participants signed written informed consent form prior to participating in the experiment.

#### Stimuli

**Figure 2:**
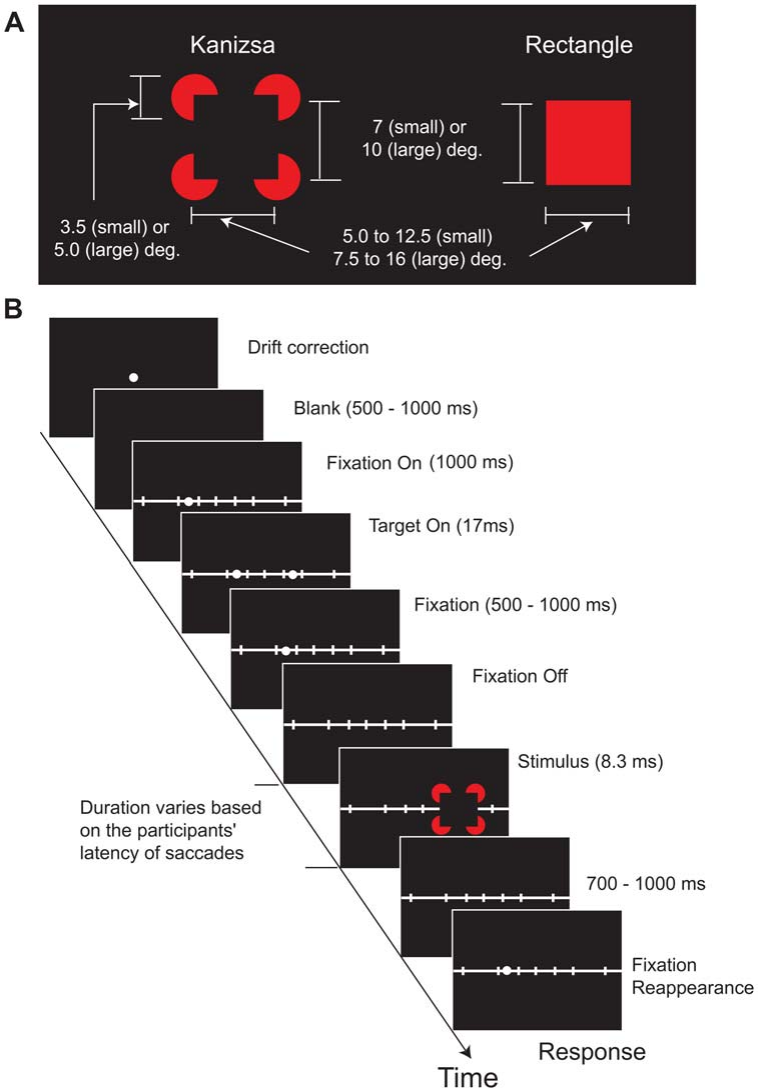
Experiment 1A: Trial design. Panel A shows examples of Kanizsa and rectangle stimuli. Panel B shows sequence of events during each trial.

Figure 2A shows examples of Kanizsa and rectangle stimuli. All stimuli were presented as red over a black background. Luminance of the figure was 3.2 cd/m^2^ and of the background was 0.32 cd/m^2^. The stimuli decayed from the screen within 1 ms. In the small figure condition, the height of all stimuli (i.e., real or illusory rectangle) was 7 degrees and the width varied from 5.0 to 12.5 degrees in six levels (5.0 6.5 8.0 9.0 11.0 12.5). In the large figure condition, the height of all stimuli was 10 degrees and the width varied from 7.5 to 16 degrees in six levels (7.5 9.0 10.5 12.0 14.0 16.0). In the small condition, the figure inducers were 3.5 degrees, whereas in the large condition the figure inducers were 5.0 degrees. Thus, a ratio of physically specified contours to the total edge length (physical and illusory), called support ratio, was 0.5 in both small and large figure conditions.

#### Apparatus

Participants were seated with their heads stabilized by a chin rest in a dimmed room. All stimuli were presented on a 21-inch CRT monitor (Calix, TOTOKU) using a 1152×864 resolution and 120 Hz refresh rate color mode. The distance between participants' eyes and the surface of the monitor was fixed to 32 cm. Eyelink eye tracker (SR research Ltd.) sampled horizontal dominant eye movement at 250 Hz with a resolution of less than 0.5 degree. Participants pressed one of the two keys on a keyboard placed in front of them to register their responses. Presentation of stimuli, collection of eye movements and registration of participants' responses was controlled by a Macintosh G4 (Apple) computer running customized Matlab based software (Mathworks) and Psychophysics and Eyelink toolboxes ([12]–[14]; see http://psychtoolbox.org/).

#### Procedure

On each trial of the saccade condition (see Figure 2B), participants were presented with and required to fixate on a white dot centered on the screen to correct for participant eye movement drifts. When participants achieved fixation on the center white dot, they pressed the Enter key and a the dot was replaced by a black screen. After a brief delay, randomly selected from between 500 to 1000 ms duration, a white filled circle (fixation point, 8 cd/m^2^) was presented 8 degrees left of the center of the screen positioned on the ruler – a white horizontal line running across the screen with seven vertical bars positioned at −20, −10, −5, 0, +5, +10, and +20 degrees relative to the center white dot. The ruler remained on the screen throughout the duration of the trial. One second later, the saccade target was presented for 17 ms (2 refresh cycles) at 8 degrees right of the center of the screen. The fixation point was turned off 500 to 1000 ms (randomly selected) after the presentation of the saccade target. The participants' task was to fixate on the fixation point, to remember the target position, and to make a saccade towards the remembered target location as soon as the fixation point disappeared from the screen. The critical figure stimulus was flashed for 8.3 ms (1 refresh cycle) centered over the saccadic target location with the onset time determined by individual participant's saccade latencies averaged over the previous five trials. The fixation point reappeared about 700 to 1000 ms later and participants were required to re-fixate on it and decide whether the vertical or horizontal dimension of the critical target figure is longer (2-AFC decision). Participants pressed the ‘F’ key if the vertical side was longer and the ‘J’ key if the horizontal side was longer. In addition, participants were instructed to press the space bar if they did not perceive the target stimulus.

To assess participants' ability to estimate the relative length of horizontal vs. vertical dimension of critical figures (rectangle, Kanizsa) while their eyes remained stationary, participants were also given fixation trials where they were only required to fixate on the fixation point without making a saccade. While they fixated on the fixation point, a critical figure was flashed on the screen at the same location as that used for the saccade condition. All other aspects of the fixation and saccade trials were the same.

If the participants' eye position was out of the 2-degrees square window surrounding the fixation point before the fixation disappeared, a warning beep was sounded to alert participants that they failed to maintain fixation; the fixation point was replaced by a black screen, the trial was discarded, and the next trial commenced. Discarded trials were re-inserted into a random position within the remaining sequence of trials. For saccade trials, we collected data until there were at least 12 valid trials for each condition (i.e., figure type x figure size x width level) that met the following criteria: saccade latency over 130 ms; saccade peak velocity less than 800 ms/s; saccade amplitude more than 13 degrees; and stimulus onset from saccade onset between −50 to −10 ms. We, therefore, avoided influences of the phosphor persistence on the monitor because even though the stimulus was presented at −10 ms before the saccade, the phosphor persistence finished before retinal images smeared by the saccade. For fixation only trials, we collected data until there were at least 12 valid trials for each condition.

The saccade and fixation trials were blocked and the order of the blocks was counterbalanced across participants. Within the blocks, the sequence of stimuli (i.e., type of figure, width level) was randomized individually for each participant and participants were given a chance to rest after each 48 trials (both valid and invalid). The experiment lasted about 3 hours including rests.

Eye movements were analyzed on-line to determine the saccade onset, saccade velocity, saccade amplitude, timing of the critical stimuli relative to the saccade onset (determined by the average saccade onset during the preceding 5 trials). The saccade onset was defined as the time when the angular velocity of the horizontal saccade exceeded 40 deg/s.

#### Data Analysis

We used bootstrapping to fit psychometric curves to each participant's data and to determine individual 50% perceptual subjective equality or PSE thresholds with 95% confidence interval limits and we used maximum likelihood estimation (MLE) to fit psychometric curves to data averaged across all participants [15], [16].

### Experiment 1b: Method

Three women and eight men (mean age 22.4 years, range 21 to 24 years) with normal or corrected-to-normal visual acuity participated in Experiment 1b. The experiment design, materials, and procedure were the same as in Experiment 1a with the following exceptions: three figures – rectangle, Kanizsa, and disk-rectangle – were used as critical stimuli and all were presented in small size only. The diameter of the disks was the same as that of the Kanizsa figure inducers. The experiment was approved by Research Ethics Committee of the Kwansei Gakuin University and all participants signed written informed consent form prior to participating in the experiment.

### Experiment 2: Method

Three women and five men (mean age 22.9 years, range 21 to 25 years) with normal or corrected-to-normal visual acuity participated in Experiment 2. The experimental design, materials, and procedure were the same as in Experiment 1a with the following exceptions: in the small size condition, the width of stimuli was fixed to 7 degrees and height varied from 5.0 to 12.5 degrees in six levels (5.0 6.5 8.0 9.0 11.0 12.5) whereas in the large size condition, the width of stimuli was fixed to 10 degrees and the height varied from 7.5 to 16.0 degrees in six levels (7.5 9.0 10.5 12.0 14.0 16.0). The experiment was approved by Research Ethics Committee of the Kwansei Gakuin University and all participants signed written informed consent form prior to participating in the experiment.

## Results and Discussion

### Experiment 1a: Results & Discussion

**Figure 3:**
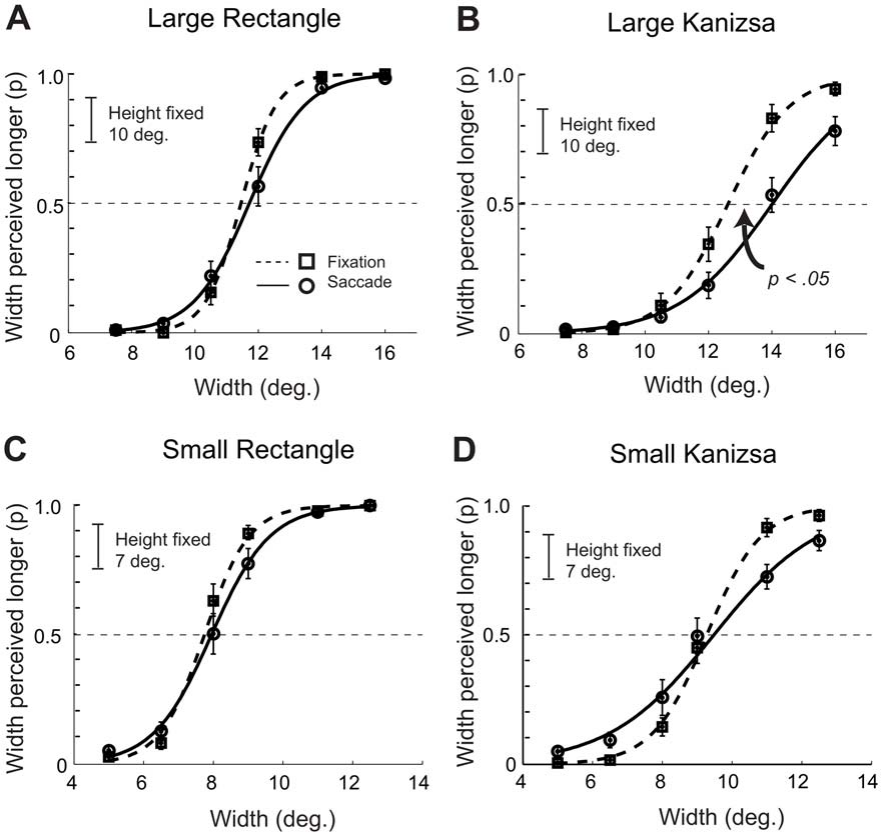
Experiment 1A: Proportion of participants reporting the width was longer than the fixed height. Vertical bars indicate SEMs.

**Figure 4:**
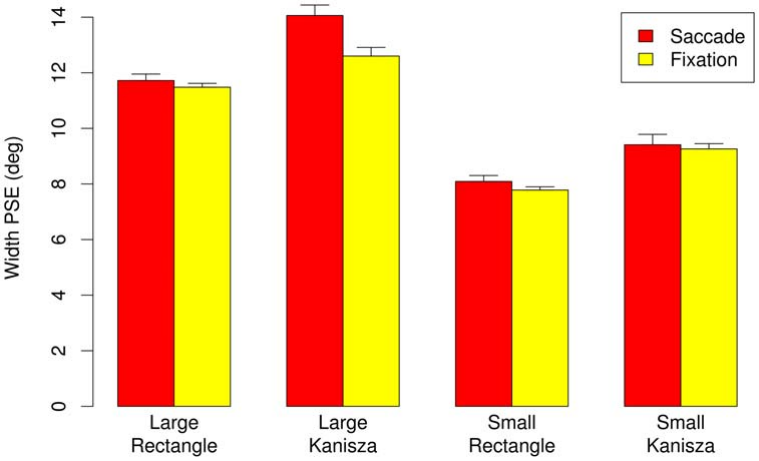
Experiment 1A: Perceived width (50% PSE thresholds) for the fixation and saccade conditions. Vertical bars indicate 95% within subject confidence intervals [17].

Figure 3 shows the psychometric curves showing the mean probability of responding that the horizontal size of the stimulus is longer than the vertical size averaged across participants, by the stimulus type (rectangle, Kanizsa), stimulus size (small, large), and eye movement condition (saccade, fixation). For large Kanizsa stimuli, psychometric curves for the saccade conditions shifted to the right relative to the fixation condition, indicating that participants perceived the width of the Kanizsa as smaller than its height. In contrast, no such shift of the psychometric curves occurred in the saccade vs. fixation condition for large rectangle stimuli, small Kanizsa stimuli, or small rectangle stimuli. Figure 4 highlights these findings; it shows perceived figure width −50% PSE thresholds averaged across the participants – by size, figure type, and eye movement condition with error bars showing 95% within subjects'confidence intervals [17].

The ANOVA over individual 50% threshold data with stimulus type (rectangle, Kanizsa), stimulus size (small, large), and eye movement (saccadic, fixation) as within subjects factors revealed significant main effects of stimulus type, F (1,12) = 53.78, MSe = 1.18, p<0.001, stimulus size, F(1,12) = 782.78, MSe = 0.49, p<0.001, eye movement, F(1,12) = 7.71, MSe = 0.99, p = 0.017, significant eye movement x stimulus size interaction, F(1,12) = 9.24, MSe = 0.26, p = 0.010, and significant three way eye movement x stimulus size x stimulus type interaction, F(1,12) = 7.69, MSe = 0.39, p = 017. No other affects approached significance. Follow up ANOVAs for small stimuli with stimulus type and eye movement as withing subjects factor revealed no significant effects or interaction except significant effect of stimulus type, F(1,12) = 66.97, MSe = 0.38, p<0.001. In contrast, Follow up ANOVAs for large stimuli with stimulus type and eye movement as withing subjects factor revealed significant stimulus type effect, F(1,12) = 30.93, MSe = 1.26, p<0.001, eye movement effect, F(1,12) = 16.56, MSe = 0.57, p = 0.002, and stimulus type by eye movement interaction, F(1,12) = 6.69, MSe = 0.71, p = 0.024. Whereas eye movement effect (saccade vs. fixation) was significant for Kanizsa figure, t(12) = 3.88, p<0.01, it was not significant for rectangle figure, t(12) = 1.03, p = 0.32.

**Figure 5:**
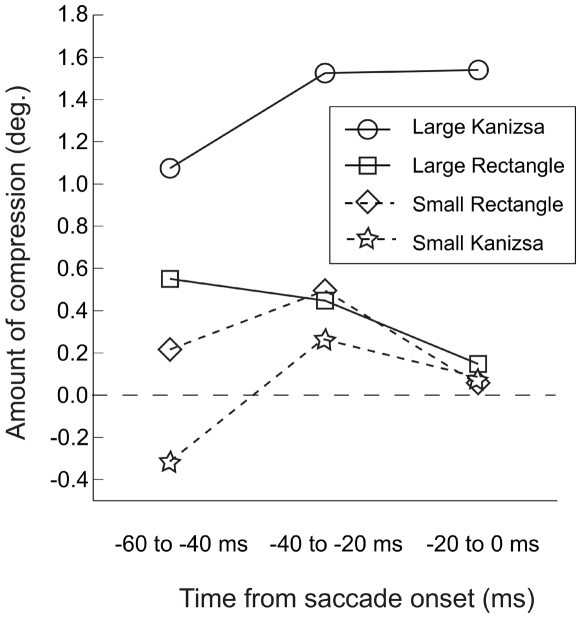
Experiment 1A: Mean amount of compression (Kanizsa-Rectangle) by figure onset time relative to saccade onset.

To determine whether the magnitude of saccadic compression increases when the Kanizsa figure is presented closer to the saccade onset, we examined the relationship between the probability that the width of the stimulus was perceived as longer than its height as a function of time between the figure onset and the saccade onset. For this purpose, we classified all trials in the saccade conditions into one of the 3 time bins – −60 to −40 ms, −40 to −20 ms, and −20 to 0 ms – depending on time elapsed between the figure and saccade onsets, calculated means for each participant and each time bin, and then calculated mean values across all participants for each time bin. Next, we used MLE methods to to find out the amount of compression in degrees for each time bin for all conditions (large Kanizsa, large rectangle, small Kanizsa, small rectangle) in the saccade condition relative to the fixation condition. Figure 5 shows the relative amount of compression for Kanizsa vs. rectangle figures by time elapsed between the figure and saccade onsets. Consistent with previous findings [3], [8], Figure 5 indicates that saccadic compression was larger for the large Kanizsa figure when the time elapsed between the figure and saccade onset was shorter. Moreover, the small Kanizsa figure was not compressed even when the critical figures were presented just before the saccade onset.

Several alternatives can be offered to explain why saccadic compression occurred only for the large Kanizsa figure. According to one class of explanations, the illusory contour induced by Kanizsa figure may prevent saccadic compression but the speed of illusory contour completion depends on the distance between the inducers allowing the re-mapping processes, running in parallel, to remap the visual space in the absence of illusory contours [18]. Alternatively, the illusory contours do not prevent saccadic compression because, for example, they operate at a higher level of the visual stream than the re-mapping processes, but saccadic compression can be prevented by reducing the distance between the inducers so that the discrete elements of the figure are seen as a whole rather than its parts by the Gestalt law of proximity.

### Experiment 1b: Results and Discussion

**Figure 6:**
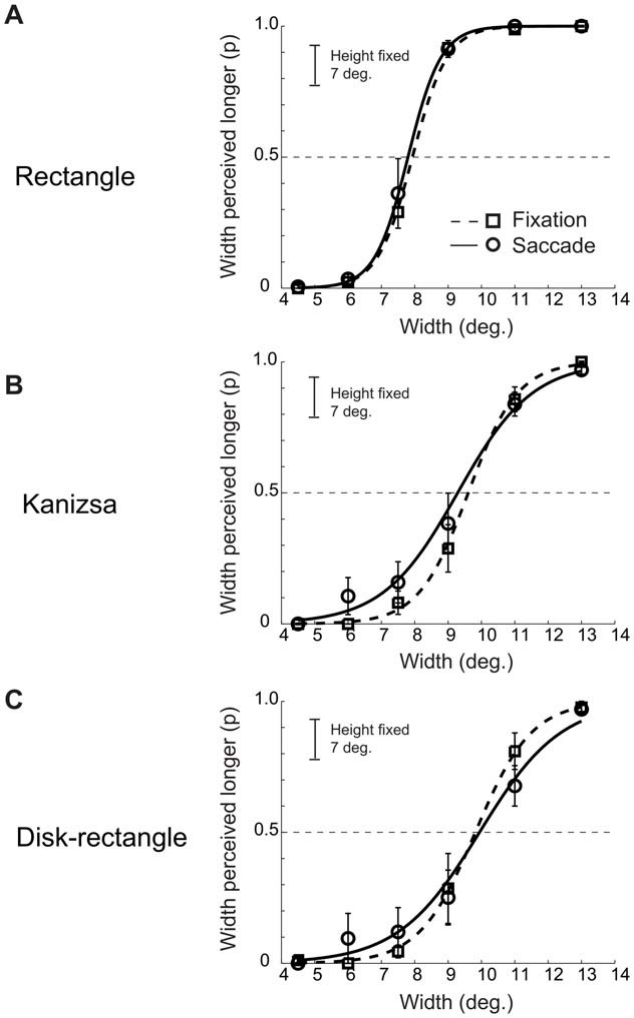
Experiment 1B: Proportion of participants reporting the width was longer than the fixed height. Vertical bars indicate SEMs.

Figure 6 shows the probability that the participants perceived the width as relatively longer than the height; it shows psychometric curves averaged across participants by critical figure (rectangle, Kanizsa, and disk-rectangle) and viewing condition (saccade, fixation). The figure shows no shifts of the psychometric curve for the saccade relative to fixation condition for any of the three figures.

**Figure 7:**
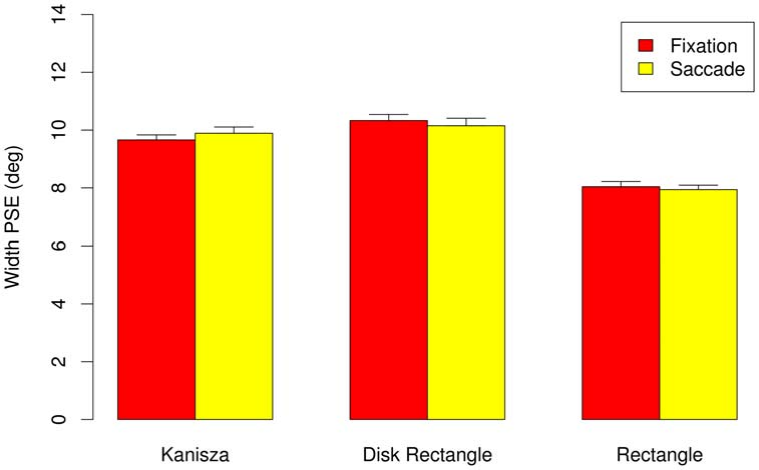
Experiment 1B: Perceived width (50% PSE thresholds) for the fixation and saccade conditions. Vertical bars indicate 95% within subject confidence intervals [17].

Figure 7 highlights these findings; it shows perceived figure width −50% PSE thresholds averaged across the participants – by size, figure type, and eye movement condition with error bars showing 95% within subjects confidence intervals [17]. The ANOVA over individual 50% threshold data with stimulus type (Kanizsa, disk rectangle, rectangle) and eye movement (saccadic, fixation) as within subjects factors. The ANOVA revealed significant main effect of stimulus type, F (2,16) = 158.75, MSe = 0.16, p<0.001, with no other effects approaching significance.

The finding that saccadic compression did not occur with either small Kanizsa or with small disk-rectangle stimuli suggests that the absence of saccadic compression for small Kanizsa figure found in Experiment 1a was not due to illusory contours but simply due to the close proximity of individual elements forming the figure. In turn, these findings suggest that element proximity determines whether saccadic compression is observed.

### Experiment 2: Results and Discussion

**Figure 8:**
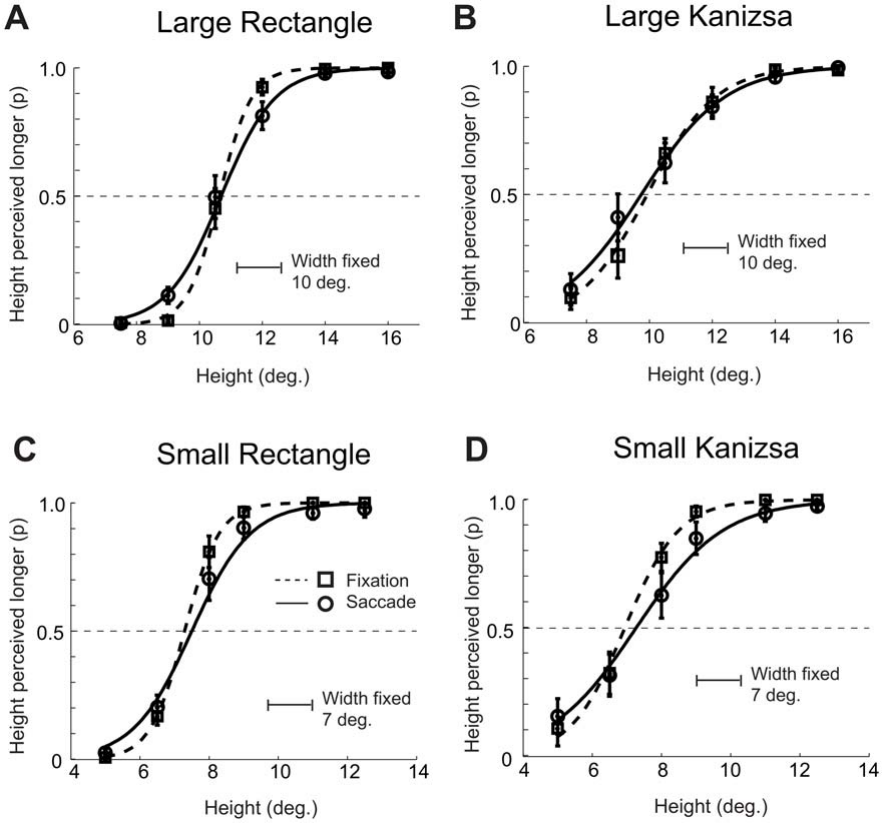
Experiment 2: Proportion of participants reporting the height was longer than the fixed width. Vertical bars indicate SEMs.

Figure 8 shows the probability that participants perceived the vertical dimension as large as the horizontal dimension; it shows psychometric curves averaged across participants in both fixation and saccade conditions, for the rectangle (top panels) and Kanizsa figure (bottom panels) and for small (left panels) and large (right panels) stimuli. In both rectangle and Kanizsa conditions, the saccade and fixation curves overlap in both small and large conditions, indicating that the presentation of the stimuli during the saccade vs. fixation condition did not affect the perceived dimensions.

**Figure 9:**
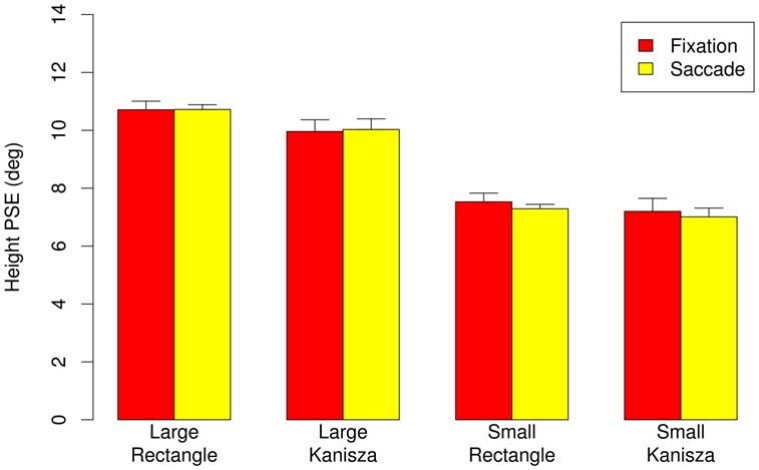
Experiment 2: Perceived height (50% PSE thresholds) for the fixation and saccade conditions. Vertical bars indicate 95% within subject confidence intervals [17].

Figure 9 shows perceived figure height −50% PSE thresholds averaged across the participants – by size, figure type, and eye movement condition with error bars showing 95% within subjects confidence intervals [17].

The ANOVA over individual 50% threshold data with stimulus type (rectangle, Kanizsa), stimulus size (small, large), and eye movement (saccadic, fixation) as within subjects factors. The ANOVA revealed no significant effects nor interaction except the main effect of stimulus size (large, small), F(1,12) = 1069.01, MSe = 0.18, p<0.001.

We also directly compared the size of the saccadic compression for the large Kanizsa figures in Experiment 1 vs. 2 and expected to obtain significant 3-way interaction between the experiment, figure, and saccadic condition. Consistent with this expectation, the ANOVA over individual 50% threshold data with the experiment (1A and 2) as between subjects factor and the figure (rectangle, Kanizsa) and saccadic condition (fixation, saccade) revealed a significant 3-way interaction between the experiment, figure, and saccadic condition, F(1,22) = 5.72, Mse = 2.65, p<0.05.

The finding that participants perceived the vertical sizes of figures as larger in Experiment 1 but not in Experiment 2 in both the fixation and saccade conditions is consistent with prior research [19]. In the classical horizontal-vertical illusion, the vertical lines are perceived as longer than the horizontal lines even though they are physically equally long. However, Armstrong and Marks [19] showed that the size of vertical-horizontal illusion is context dependent. When the horizontals were longer than the verticals the size of the horizontal-vertical illusion was large. In contrast, when the verticals were longer than the horizontals, the size of the horizontal-vertical illusion was very small, only a few percent. Similarly, in our Experiment 1 the horizontals were more often longer than the verticals and we observed substantial horizontal-vertical illusion in both the fixation and saccade conditions. In contrast, in our Experiment 2, the verticals were more often longer than the horizontals and we observed no or only minimal horizontal-vertical illusion effects in Experiment 2.

The null effect of the fixation vs. saccade condition could be due to (1) no horizontal and no vertical compression or (2) both vertical and horizontal compression occuring simultaneously. However, the data from Experiment 1 make the second interpretation extremely unlikely. Experiment 1 revealed that the saccadic compression occurred only for large Kanizsa (10 degrees in height) and that the size of the compression increased with the horizontal width, that is, the compression increased as the horizontal distance between the inducers increased from 5.5 to 16 degrees. Specifically, no horizontal compression occurred when the horizontal width was smaller than 10 to 11 degrees and the horizontal compression was the largest when the horizontal distance between the inducers was large, 14 to 16 degrees. Given that the horizontal distance between the inducers in Experiment 2 was only 10 degrees, we can assume that no horizontal compression occurred in Experiment 2. In turn, given that there were no differences between the fixation and saccade conditions and the vertical distances between the inducers ranged up to 16 degrees in Experiment 2, the data indicate that the absence of the null effect of the fixation vs. saccade condition indicates that there was no vertical compression. Moreover, this interpretation is consistent with the findings of Kaiser and Lappe [9] study showing that the vertical compression does not occur for dots flashed within 20 degrees of the fixation point.

In combination, these results indicate that the vertical compression does not occur even with the presentation of the complex stimuli such as rectangles and Kanizsa figures.

### General Discussion

Our findings unequivocally show that large but not small Kanizsa figures are perceived as compressed in the horizontal dimension and that such compression is stronger when the Kanizsa figure is presented closer to the saccade onset. In contrast, small Kanizsa figures and rectangle figures, regardless of size, are perceived as undistorted with no horizontal compression. Finally, we found no evidence of saccadic compression for our control stimuli, rectangles, in either the horizontal or vertical dimension.

Thus, using different methodology, we have obtained results consistent with Sogo and Osaka's [8] conclusions that the Kanizsa figure is perceived as horizontally compressed and that the magnitude of such saccadic compression is dependent upon the distance between individual elements of the figure. However, our results extend the previous findings in several important ways. Most importantly, we show for the first time that compression of the Kanizsa figure is larger in the horizontal than in the vertical dimension. Sogo and Osaka examined only perceived compression in the horizontal dimension, and thus, their results are consistent with the possibility that Kanizsa figures are compressed in both horizontal and vertical dimensions and do not exclude the possibility that vertical compression is larger. The results of our experiments are unequivocal: horizontal compression is larger than vertical compression.

In our experiments, participants make decisions about the ratio of horizontal vs vertical dimensions in Experiment 1 and the ratio of vertical vs. horizontal dimensions in Experiment 2, while we varied the horizontal and vertical dimensions of stimuli in Experiments 1 and 2, respectively. Accordingly, someone may argue that our finding of horizontal compression in Experiment 1 but not in Experiment 2 could be due to a bias effect rather than to saccadic compression. In any case, more studies are needed to examine whether horizontal vs. vertical compression occurs for different stimuli and/or stimuli with different ranges of dimensions (i.e., larger or smaller than in our experiments). However, we believe our findings reflect true saccadic compression in Experiment 1 for the following reasons. First, any perceptual biases (for example, horizontal vs. vertical illusion effects) not related to saccadic movement are controlled by our fixation conditions. Second, to explain the findings, any such saccade related bias would need to operate only on large Kanizsa figures but not on control rectangle figures or small Kanizsa figures that were not subject to any saccadic compression. Third, one may argue that the results could be due to vertical extension rather than horizontal compression of Kanizsa figure. While this is a possibility, it is unlikely because no one has ever reported vertical nor horizontal saccadic extension in prior research. Fourth, our findings are consistent with Sogo and Osaka's [8] finding of saccadic horizontal compression for Kanizsa figures that were slightly larger than our large figures. Fifth, our findings are consistent with a wealth of prior research demonstrating horizontal compression for figures that are seen as disparate collections of elements and no horizontal compression for figures seen as unitized wholes (e.g., our control rectangle figures). Similarly, in Experiment 2, the null effect of the fixation vs. saccade condition could be due to (1) no horizontal and no vertical compression or (2) both vertical and horizontal compression occurring simultaneously. However, the second interpretation is unlikely. Experiment 1 revealed that the saccadic compression occurred only for large Kanizsa (10 degrees in height) figures and that no horizontal compression occurred when the horizontal width was smaller than 10 to 11 degrees. Given that the horizontal distance between the inducers in Experiment 2 was only 10 degrees, we can assume that no horizontal compression occurred in Experiment 2 and thus, that no differences between the fixation and saccade conditions indicate that there was no vertical compression. Moreover, this interpretation is consistent with the findings of Kaiser and Lappe [9] (see above).

Our findings showing no horizontal compression for our control figures – rectangles – is consistent with the finding by Matsumiya and Uchikawa [6] showing no horizontal compression with rectangle stimuli using a sequential horizontal comparison method. On each trial, Matsumiya and Uchikawa presented participants with a target rectangle just prior to a saccade onset followed by a probe rectangle shortly after the saccade offset. The probe rectangle was either shorter or longer than the target rectangle and participants were required to decide whether the probe was shorter or longer than the target. In turn, Matsumiya and Uchikawa's findings combined with our findings suggest that saccadic compression does not occur in horizontal or vertical dimensions for rectangle stimuli. Thus, these findings contrast sharply with Sogo and Osaka's [8] findings showing substantial saccadic horizontal compression for rectangle stimuli in their experiment.

One explanation for these contrasting findings is that the design features of Sogo and Osaka's method are responsible for the unexpected horizontal compression of the control rectangle figures in their experiment. First, as pointed out in the introduction, Sogo and Osaka's data may underestimate or overestimate the degree of horizontal compression depending on the particular task strategy participants choose on any given trial – adjusting the length of the probe to the *length of remembered figure* or adjusting the length of the probe so that the probe matches the *shape of the remembered figure*. Second, in Sogo and Osaka's experiment, participants could make a judgment only after appearance of a probe more than 1.5 s after a stimulus presentation, and thus, their data may show large compression for rectangles not because of the saccadic compression but because rectangle stimuli are *remembered* rather than perceived as compressed [10]. Third, a horizontal-vertical illusion that makes horizontal dimension appear shorter than vertical dimension may have inflated the size of horizontal compression for both the rectangle and Kanizsa figures in Sogo and Osaka's study [19], [20]. Fourth, Sogo and Osaka included only three participants in each of their experiments, and thus, their results need not generalize to larger samples. Thus, the substantial compression of rectangle figures observed by Sogo and Osaka suggests that their Kanizsa figure compression data overestimate the magnitude of saccadic compression simply due to the method features, especially strategy differences and memory effects. In turn, our findings highlight the importance of studying various perceptual phenomena by a variety of methods.
